# The Proatherogenic Effect of Chronic Nitric Oxide Synthesis Inhibition in ApoE-Null Mice Is Dependent on the Presence of PPAR****α****

**DOI:** 10.1155/2014/124583

**Published:** 2014-01-22

**Authors:** Michal Vechoropoulos, Maya Ish-Shalom, Sigal Shaklai, Jessica Sack, Naftali Stern, Karen M. Tordjman

**Affiliations:** The Institute of Endocrinology, Metabolism, and Hypertension, Tel Aviv-Sourasky Medical Center, The Sackler Faculty of Medicine, Tel Aviv University, 6 Weizmann Street, 64239 Tel Aviv, Israel

## Abstract

Inhibition of endothelial nitric oxide synthase (eNOS) accelerates atherosclerosis in ApoE-null mice by impairing the balance between angiotensin II (AII) and NO. Our previous data suggested a role for PPAR**α** in the deleterious effect of the renin-angiotensin system (RAS). We tested the hypothesis that ApoE-null mice lacking PPAR**α** (DKO mice) would be resistant to the proatherogenic effect of NOS inhibition. DKO mice fed a Western diet were immune to the 23% worsening in aortic sinus plaque area seen in the ApoE-null animals under 12 weeks of NOS inhibition with a subpressor dose of L-NAME, *P* = 0.002. This was accompanied by a doubling of reactive oxygen species (ROS-) generating aortic NADPH oxidase activity (a target of AII, which paralleled Nox1 expression) and by a 10-fold excess of the proatherogenic iNOS, *P* < 0.01. L-NAME also caused a doubling of aortic renin and angiotensinogen mRNA level in the ApoE-null mice but not in the DKO, and it upregulated eNOS in the DKO mice only. These data suggest that, in the ApoE-null mouse, PPAR**α** contributes to the proatherogenic effect of unopposed RAS/AII action induced by L-NAME, an effect which is associated with Nox1 and iNOS induction, and is independent of blood pressure and serum lipids.

## 1. Introduction

Expressed in all the cellular components of the vascular wall, and present in the atherosclerotic plaque, the precise role of the peroxisome proliferator-activated receptor alpha (PPAR*α*) in atherogenesis is still controversial. Its known effect on lipoprotein metabolism, and mostly surrogate endpoints derived from animal studies, helped shape the view that its activation confers protection against atherosclerosis (for review [1]). Large clinical trials designed to assess the potential of fibrates to reduce the rate of cardiovascular endpoints have, however, reached mixed results, suggesting that benefit may be restricted to subsets of subjects with defined lipoprotein abnormalities [2–4]. We previously reported that ApoE-null mice lacking PPAR*α* were resistant to diet-induced atherosclerosis, despite exhibiting the worsened lipid profile expected from the absence of PPAR*α*. In addition, the double knockout mice had also a somewhat lower blood pressure [5]. Although by itself this reduction could not explain the protection from atherosclerosis, it suggested that PPAR*α* could affect a system central to both atherogenesis and blood pressure regulation. In this respect, a natural candidate is the renin-angiotensin system (RAS). We subsequently showed that ablation of PPAR*α* totally abolished hypertension and greatly reduced diet-induced atherosclerosis in the Tsukuba hypertensive mouse, a model of angiotensin II (AII-) mediated hypertension and atherosclerosis due to the transgenic expression of the human renin and angiotensinogen genes. In this model, absence of PPAR*α* markedly reduced the level of circulating kidney-derived human renin (the rate-limiting step of the RAS), and also that of human renin secreted in the medium by aortic smooth muscle cell primary cultures established form these mice, suggesting that some of the vascular protection could stem from downregulation of the tissue RAS in the vessel wall [6].

A delicate balance between AII and nitric oxide (NO) in vascular health has been well recognized [7]. AII elevates blood pressure, reduces the generation of NO, increases the production of reactive oxygen species (ROS) mostly through nicotinamide adenine dinucleotide phosphate (NADPH) oxidase, and thus promotes inflammation and atherosclerosis. In contrast, endothelium-derived NO lowers blood pressure, reduces the accumulation of ROS, suppresses inflammation, and ultimately limits atherosclerosis. Thus any event that might downplay the NO side of this balance incurs the potential of promoting atherosclerosis. Indeed, it has been demonstrated that genetic or pharmacologic ablation of NO synthase (NOS) accelerates atherosclerosis in the ApoE-null mouse [8, 9].

We hypothesized that as PPAR*α* appears to be required for the full deleterious effect of the RAS, the double ApoE/PPAR*α* knockout (DKO) mouse should be resistant to the worsening of atherosclerosis induced by chronic inhibition of endothelial NOS (eNOS) activity by a subpressor dose of N_*ω*_-nitro-L-arginine methyl ester hydrochloride (L-NAME). In the current report we show this to be the case, and we also point at two main culprits in the PPAR*α*-dependent proatherogenic effect of eNOS inhibition, namely, Nox1 and iNOS.

## 2. Methods

### 2.1. Animals and Study Design

ApoE-null mice maintained at the Tel Aviv-Sourasky Medical Center animal facility were crossbred with PPAR*α*-null mice; both lines were on the C57Bl/6 genetic background following extensive backcrossing. Identified by genotyping (http://jaxmice.jax.org/pub-gi/protocols/protocols.sh?objtype=protocol&protocol_id=221), F2 doubly transgenic founders were then used to create the DKO line. In these experiments ApoE-null and DKO mice were used under the same protocol.

At the age of 4 weeks, half the animals were given a subpressor dose of L-NAME (5 mg/L), an inhibitor of NOS, in the drinking water (Sigma-Aldrich Cat number N5751). This dose was based on that given to rats, which was shown to be devoid of pressor effects, while it still reduced both plasma and urinary NO production [10, 11]. There were thus 4 experimental groups, each comprising approximately 20 mice. At the age of 8 weeks, noninvasive basal blood pressure was obtained as described [12], and animals were switched to a high fat Western diet (Teklad diet 88317, Harlan, Madison, WI) for 8 weeks. L-NAME administration was continued throughout the experiment.

At the end of the experiment, blood pressure was recorded again. After a 4 h fast, under light isoflurane anesthesia, blood samples were obtained from the retro-orbital plexus for biochemical determinations. Animals were sacrificed with a lethal dose of isoflurane. All experimental protocols were carried out after obtaining the authorization of the institutional committee for experiments in laboratory animals and conformed to the NIH Guide for the Care and Use of Laboratory Animals [13].

### 2.2. Biochemical Determinations and Fast Protein Liquid Chromatography (FPLC) Analysis of Lipoproteins

Serum biochemistry was assessed on an Advia 1650 autoanalyzer (Siemens AG, Germany). In addition, the various lipoprotein fractions were also analyzed by FPLC. For this procedure 4 samples from each animal group, each sample representing pooled plasma from 2 mice and diluted 1 : 1 v/v in buffer, were first filtered through a 0.45 *μ* filter to remove chylomicrons. Samples were loaded on a superpose-6 column (GE Pharmacia) and separated by size exclusion into 41 fractions. VLDL particles were typically collected between tubes 15–19, LDL between tubes 21–27, and HDL between tubes 29–37. Following separation, the cholesterol concentration of each fraction was determined in a colorimetric reaction (cholesterol reagent, Roche) on a microplate and read on an ELISA reader (Cobas, Roche) at 495 nm.

### 2.3. Heart and Aorta Processing and Atherosclerosis Analysis

The aortas were snap-frozen for RNA isolation and for NADPH oxidase activity determination. The hearts were sectioned through the ventricles; the upper third including the aortic root was embedded in OCT and frozen until analysis.

For assessment of atherosclerosis, 10 *μ*m cryostat sections of the hearts encompassing the area of the aortic sinus were collected and stained with Oil-Red-O. Quantification of the plaques was performed using a digital imaging processing program (NIS element Br 3.0 imaging system) (Nikon Instruments Europe B.V., The Netherlands), as described [12].

### 2.4. NADPH Oxidase Activity Assessment

NADPH oxidase activity was measured in aortas in an in-house lucigenin-enhanced chemoluminescent assay as follows. Aortas were thoroughly cleaned from adjacent fat and connective tissue, isolated in ice-cold Krebs-Hepes buffer, pH 7.4, and snap-frozen in liquid N_2_ until assayed at which time they were thawed in ice-cold KHB and kept on ice. Under binocular magnification, aortas were meticulously cleaned from all adjacent tissues and cut into 3–5 mm rings. They were subsequently incubated at 37°C for 45 min in prewarmed KHB. Each ring was then placed in an optical plate well in 175 *μ*L of KHB containing freshly made NADPH (Sigma-Aldrich Cat. number N6505) to yield a final reaction concentration of 100 *μ*M. The reaction started after the automatic injection of 25 *μ*L of lucigenin (Sigma-Aldrich Cat number M8010) to give a final concentration of 5 *μ*M. Luminescence was measured every 5 seconds for 1 minute on a LUMIstar Galaxy luminometer (BMG Labtech, Offenburg, Germany). After the subtraction of background (recorded in the absence of tissue), the average luminescence for each sample was adjusted for the dried weight of the ring, and the mean NADPH oxidase activity of each aorta (6–8 rings) was expressed as relative luminescence units·mg^−1^·min^−1^. Under the experimental conditions, the luminescence was specific for NADPH oxidase as the fluorescence in the absence of added substrate (NADPH) was negligible.

### 2.5. Aortic Gene Expression Studies

After RNA isolation (TRIzol, Invitrogen, Life Technology, Carlsbad, CA) and reverse transcriptase synthesis of cDNA, the level of expression of several relevant genes was assessed on a StepOne Real-Time System (Applied Biosystems, Life Technology).

The following TaqMan gene expression assays on demand were used: renin: MM02342887_MH; angiotensinogen: AGT-MM00599662_M1; angiotensin converting enzyme 1: ACE1-MM00802048_M1; angiotensin II type 1 receptor: AT1-R-AGTR1a MM00616371_M1; endothelial nitric oxide synthase: eNOS-MM00435217_M1; inducible NOS: iNOS-MM01309897-M1, with HPRT as the endogenous gene MM00446968_M1. In addition, aortic expression of monocyte chemotactic protein 1 (MCP1), and that of the NADPH oxidase genes Nox1, Nox2, and Nox4, was assessed semiquantitatively. The level of aortic expression of the following genes was determined by semiquantitative PCR in the linear range of the reactions, using beta-actin as the housekeeping, and the following forward and reverse primers:

 MCP1: 5′-CATTCACCAGCAAGATCC-3′;
 5′-CTCATTTGGTTCCGATCCAG-3′;
 Nox1: 5′-ATATTTTGGAATTGCAGATGAACA-3′;
 5′-ATATTGAGGAAGAGACGGTAG-3′;
 Nox2: 5′-CTTGGGTCAGCACTGG-3′;
 5′-TTCCTGTCCAGTTGTCTTCG-3′;
 Nox4: 5′-TTGTCTTCTACATGCTGCTG-3′;
 5′-AGGCACAAAGGTCCGHAAAT-3′;
 Beta actin: 5′-GACTACCTCATGAAGATCCTG-ACC-3′;
 5′-TGATCTTCATGGTGCTAGGAGCC-3′.

All reactions were carried out with a 2 mM MgCl_2_ final concentration (except for Nox1 that required 4 mM), using the Promega GoTaq Green Master Mix (Promega Corp. Madison, WI). PCR products were size-separated by electrophoresis in an ethidium bromide-containing 2% agarose gel. The band fluorescence intensity was captured on the 202D Bio-Imaging System (Dinco, Rhenium, Jerusalem, Israel) and analyzed with TINA software (Raytest, Straubenhardt, Germany).

### 2.6. Statistical Analysis

Data are expressed as mean ± SE. Groups were compared by parametric ANOVA followed by posttests. A repeated measure ANOVA was used for parameters obtained at baseline and at the end of the experiment. When comparison between the 4 groups was deemed unnecessary, Student's *t*-test was used. Correlations between parameters were established using linear regression or Spearman rank correlation. Statistical significance was assumed for *P* < 0.05.

## 3. Results

### 3.1. Animals' Weight, Blood Pressure, Serum Biochemistry, and FPLC of Lipoproteins

**Figure 1:**
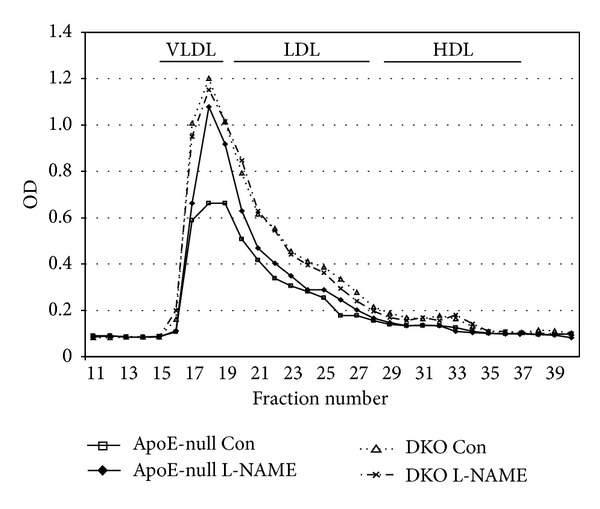
Lipoprotein FPLC analysis. Each curve represents the average of 4 samples, pooled from the sera of 2 mice each (error bars omitted for clarity). L-NAME increased VLDL cholesterol in the ApoE-null mice to the level seen in the DKO. DKO mice were not affected and maintained significantly higher LDL under all conditions (*P* < 0.01 for area under the curve, AUC).

Deliberately given at a subpressor dose, L-NAME had indeed no effect on animals' blood pressure. All animals were normotensive both at baseline and after 8 weeks of high fat feeding, independently of treatment and despite increased adiposity in the DKO animals already detected at baseline (Table 1). As expected from the role of PPAR*α* in lipoprotein metabolism, cholesterol levels were twice as high, and triglycerides were 3 times higher in the DKO mice than in the ApoE-null mice following the high fat feeding period. However, L-NAME increased cholesterol by another 39% and triglycerides by more than 50% in the ApoE-null mice, while it was without any effect in the DKO. Such a rise essentially brought the cholesterol to equal levels in both lines (Table 1). FPLC analysis followed by cholesterol determination in the various fractions subsequently confirmed that the elevation caused by L-NAME was essentially limited to very low density lipoproteins (VLDL). Low density lipoprotein (LDL) cholesterol, however, unaffected by L-NAME remained significantly higher in the DKO (Figure 1).

### 3.2. DKO Mice Have Less Atherosclerosis and Are Immune to the Proatherogenic Effect of L-NAME

**Figure 2:**
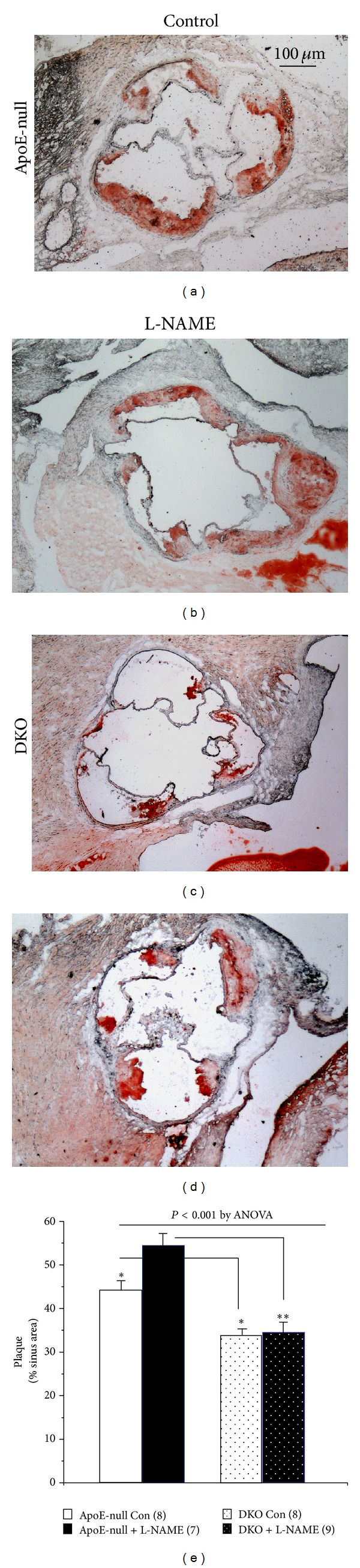
Atherosclerosis at the aortic sinus. Representative photographs of the oil-red-O-stained lesions ((a)–(d)), and after quantification (e), mice number in parentheses. Atherosclerosis was 23% lower in the DKO control mice (c) versus the ApoE-null (a), *P < 0.05. L-NAME increased the extent of the plaque by 23% in the ApoE-null mice, ((a), (b), and (e)), *P < 0.05, but had no effect in the DKO ((c), (d), and (e)), resulting in a 37% greater plaque area in the treated ApoE-null mice versus the treated DKO animals, **P < 0.005.

Confirming our earlier observations [5], the DKO control mice developed less atherosclerosis at the aortic sinus than their ApoE-null counterparts despite having a worse lipoprotein profile. Indeed, after 8 weeks on the Western diet, the atherosclerotic plaque encompassed 44.1% of the sinus area in the ApoE-null mice, yet only 33.8% in the DKO, a 23% difference, *P* = 0.01, (Figures 2(a), 2(c), and 2(e)). The DKO mice were also immune to the proatherogenic effect of blocking NO generation with L-NAME, as the plaque covered 34.4% of the sinus in the treated animals (Figures 2(d) and 2(e)). In contrast, L-NAME treatment increased the extent of the plaque in the ApoE-null mice by another 23% compared to control, to cover 54.3% of the sinus area (Figures 2(b) and 2(e); *P* < 0.05 compared to control), thereby creating a plaque area that was 37% larger than that measured in the treated DKO (*P* = 0.002).

### 3.3. Aortic NADPH Oxidase Activity Is Induced by L-NAME Only in ApoE-Null Mice and Correlates with NOX-1 Expression and with Atherosclerosis

**Figure 3:**
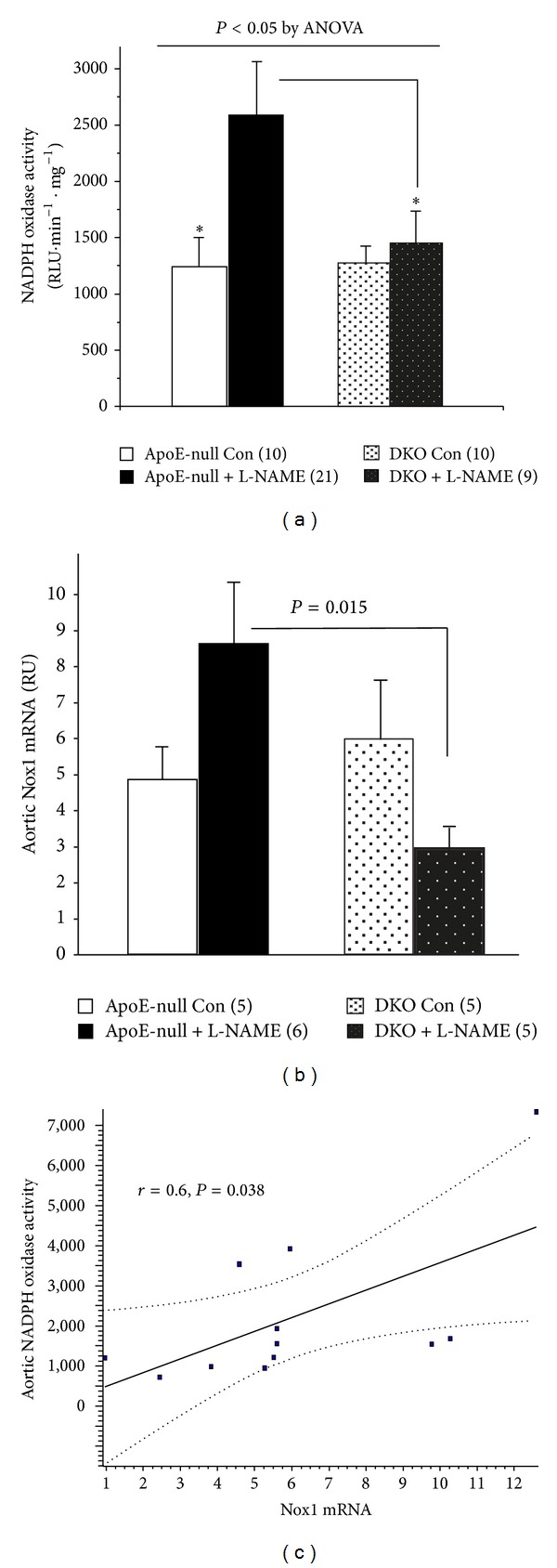
Aortic NADPH oxidase correlates with Nox1. (a) DKO mice are immune to the significant (*P < 0.05) induction of NDAPH oxidase activity induced by L-NAME in the ApoE-null mice (mice number). (b) Relative expression of Nox1 mRNA (adjusted for actin) in mice aortas (mice numbers), which parallels NADPH oxidase activity, and is significantly correlated to it in a subset of mice in which both measurements were performed (c).

NADPH oxidase, the main ROS generating system, is a major player in the initiation and development of atherosclerosis. We assessed its activity in the entire aorta. NADPH oxidase activity was similar in control, high fat-fed animals in both lines. However, inhibition of NO generation by L-NAME doubled the activity in the ApoE-null mice (*P* < 0.05 versus control) but was without any effect in the DKO (Figure 3(a)). An insight into the relevance of this system was the finding that the extent of atherosclerosis was also associated with the degree of NADPH oxidase activity (*r* = 0.48, *P* = 0.03).

As several isoforms of NADPH oxidase are expressed in the vasculature, we questioned which form might contribute to the activity measured. This was addressed in part by examining the expression of Nox1, Nox2, and Nox4 in the aorta. While the level of Nox1 mRNA in the control was similar in the ApoE-null mice and the DKO, much like the activity level, L-NAME treatment induced an 80% increase in the expression of Nox1 in the ApoE-null mice, whereas it tended to suppress it in the DKO (*P* = 0.07 versus control), leaving it at a mere 1/3 of that measured in the ApoE-null animals (Figure 3(b)). Although Nox2 was not augmented by L-NAME in the ApoE-null mice, the level observed under treatment in the DKO aortas was about half that seen in the ApoE-null animals (*P* = 0.02). Nox4 expression on the other hand was identical in both lines and was not affected by L-NAME treatment (not shown). In fact, the significant positive correlation found between NADPH oxidase activity and the level of expression of Nox1 mRNA in the aorta (Figure 3(c)) suggests this isoform of NADPH oxidase, a well-recognized AII target, is driving the increase in activity measured under L-NAME in the ApoE-null mice.

### 3.4. Aortic Angiotensinogen and Renin Are Induced by L-NAME in Apo-E Null Mice but Not in the Absence of PPAR*α* (DKO Mice)

We had previously reported that the attenuation of atherosclerosis in the DKO was accompanied by a sustained reduction in the aortic expression of MCP1, compared to that seen in the ApoE-null mice, and that this effect was dependent on the presence and the activation of PPAR*α*. A potent proinflammatory chemokine, MCP1, is induced by AII and has been implicated in the development of atherosclerosis in the ApoE-null mouse [14]. We therefore questioned whether it was involved in the observed differential effect of L-NAME on atherosclerosis. As a whole, MCP-1 expression was greatly reduced in the DKO mice, but it was not affected by L-NAME-induced NOS inhibition. Like MCP1, the aortic expression of the ACE-1 mRNA was considerably lower in the DKO but unaffected by L-NAME in either line. In contrast, tissue expression of renin and angiotensinogen more than doubled with L-NAME treatment in ApoE-null mice with the wild type PPAR*α* gene but not in the DKO mice (Table 2). The absence of PPAR*α* was then linked to lesser expression of aortic ACE and with the absence of aortic renin and angiotensinogen induction by L-NAME. Taken together these changes would favor more tissue AII generated under all experimental conditions in the ApoE-null mice aortas.

### 3.5. Aortic iNOS Robustly Correlates with Atherosclerosis

**Figure 4:**
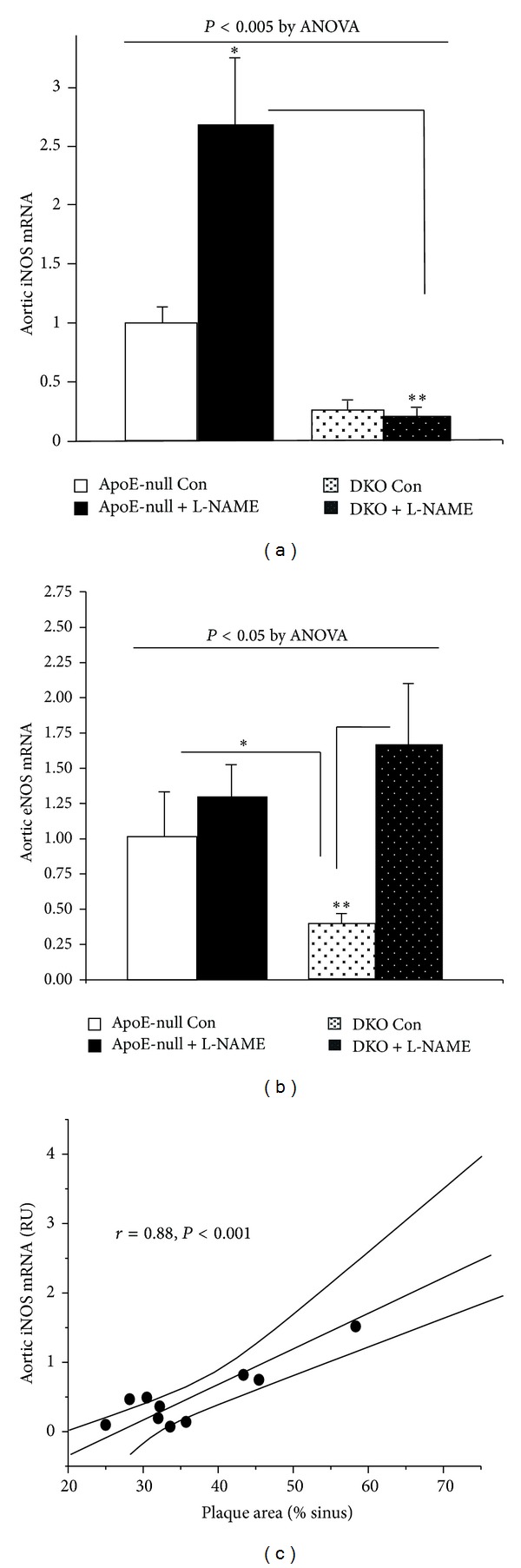
Aortic iNOS is induced by L-NAME in ApoE-null mice and correlates with atherosclerosis. Effects are expressed relative to the control ApoE-null mice. (a) iNOS expression by real-time PCR indicates a 4-fold excess in control ApoE-null versus DKO (*P < 0.05) and a tenfold difference after L-NAME (**P < 0.01), number of mice used in the experiment: 9 apoE-null control: 7 apoE-null L-NAME, 8 DKO control, and 8 DKO L-NAME. (b) eNOS is significantly increased by L-NAME in the DKO but not in the ApoE-null mice, *n* = 5 animals in each group. (c) Significant positive correlation between the extent of the plaque and iNOS expression.

Contrarily to eNOS whose net effect is to supply NO for vasodilation, antithrombotic, and antiatherogenic purposes, iNOS, not normally significantly active in the vascular wall, is induced by inflammatory cytokines and ROS. The abundant NO production that it then generates contributes to the formation of peroxynitrite, increasing the oxidative stress and rendering eNOS dysfunctional by uncoupling its activity, ultimately promoting inflammation and atherosclerosis. In view of the heightened expression of MCP1, and the induction of NADPH oxidase activity in the ApoE-null mice, conditions conducive to the induction of iNOS, we assessed its expression in the mice aorta and expected to see a greater level in the ApoE-null mice. In control ApoE-null mice the level of iNOS mRNA was 4 times higher than that in the untreated DKO mice. L-NAME treatment further increased iNOS 2.7-fold in the ApoE-null mice, while in contrast it had no effect on iNOS in the DKO mice. This resulted in ~10 fold higher expression of aortic iNOS in L-NAME-treated ApoE null mice compared to L-NAME-treated DKO (Figure 4(a)). Further support for the pathophysiologic significance of this observation comes from the strong correlation between the extent of atherosclerosis and the level of aortic iNOS, *r* = 0.88, *P* < 0.001 (Figure 4(c)). Control ApoE-null mice had a higher degree of expression of aortic eNOS than the DKO mice; however, this failed to increase under L-NAME treatment, while it more than tripled in the DKO (Figure 4(b)).

Finally, in a multiple regression analysis that included the variables shown to be correlated to the extent of the plaque by univariate analysis (MCP-1, NADPH oxidase activity, and the level of iNOS mRNA), NADPH oxidase activity along with iNOS alone predicted 86% of the atherosclerosis under the study conditions, *P* < 0.01. No other variable studied had any significant impact in predicting the extent of atherosclerosis. Notably, in this paradigm, the extent of atherosclerosis was unrelated to the severity of the hyperlipidemia.

## 4. Discussion

The salient finding of the current study is that absence of PPAR*α* gene prevents the aggravation of diet-induced atherosclerosis elicited by L-NAME in the ApoE-null mouse *in vivo*, independently of blood pressure or serum lipid alterations. These results extend and reinforce our previous reports that the absence of PPAR*α* is protective of atherosclerosis driven by ApoE-null/high fat diet status [5] as well as by overexpression of the RAS in the Tsukuba hypertensive mouse [6]. That the absence of PPAR*α* also prevents L-NAME-induced atherosclerosis on the genetic background of ApoE-KO, reemphasizes the role of this gene in the development of atherosclerosis driven by several different triggers.

An important aspect of our study is that we employed 20 times lower than that reported in various rodent models of atherosclerosis in which this agent was delivered in the drinking water as was done in the current study [8]. None of these studies presented hard data regarding blood pressure; at the most, they stated that treatment had no effect. Thus it is hard to exclude that the accelerated atherosclerosis reported under L-NAME was not also due to an unappreciated increase in blood pressure and shear stress. In contrast, as per our design, the dose chosen for L-NAME (approximately 1.5 mg·kg^−1^·d^−1^) resulted in no elevation of blood pressure in either strain, while it has been shown to effectively reduce NO production [10, 11]. Thus, by preventing L-NAME-induced hypertension and maintaining identical blood pressure throughout the study in all animal groups, we have excluded the possibility that our findings might be explained by higher blood pressure and/or shear stress.

Complementary to the exclusion of the role of L-NAME-induced hypertension in our model are the observed changes in serum lipids, which likewise cannot explain the aggravation of atherosclerosis in L-NAME treated mice. L-NAME was previously reported to elevate circulating lipids [15–17] due to increased triglyceride synthesis through induction of hepatic phosphatidate phosphohydrolase (an enzyme essential in triglyceride synthesis) and decreased oxidation due to suppression of carnitine palmitoyltransferase I (CPT-1), and elevation of cholesterol secondary to lower bile acid synthesis due to suppression of hepatic cholesterol 7 alpha-hydroxylase (CYP7A1), the latter two genes being known targets for PPAR*α* [18, 19]. Yet, in the present study, DKO mice had, as expected, higher circulating lipid levels, and while L-NAME did induce an increase in lipid levels in the ApoE-null mice, it merely brought circulating lipids to the same level seen in L-NAME-treated DKO mice. Hence, the protection from the L-NAME-related acceleration of atherosclerosis seen in the DKO cannot be ascribed to circulating lipids, which calls for the examination of other possibilities.

NADPH oxidase, the main superoxide ROS generator in the vasculature, is a target of AII. Its activation causes a burst of ROS generation that ultimately brings about endothelial dysfunction, uncouples eNOS, thereby limiting NO availability, which then initiates more superoxide and reactive nitrogen species production. The level of NADPH oxidase activity in the control mice of both lines after 8 weeks on the Western diet was identical. However, upon concomitant L-NAME treatment, the level of activity doubled in the ApoE-null mice but barely changed in the DKO. As other potential stimuli of NADPH oxidase activation such as hyperglycemia, LDL cholesterol, and shear stress can be excluded to account for this difference, it is conceivable that upregulation of NADPH oxidase under low dose L-NAME treatment is dependent on the presence of PPAR*α* and could reflect unopposed AII action.

Nox1, Nox4, and Nox2 are expressed in the vasculature. Nox1 is constitutively expressed at low levels in the endothelium and at higher levels in vascular smooth muscle cells (VSMC). It is induced in both cell types in culture by AII [20, 21]. Moreover, and most relevantly, genetic ablation of Nox1 was shown to greatly reduce the extent of diet-induced atherosclerosis in ApoE-null mice [22]. Both Nox2 and Nox4 are felt to be implicated in cardiovascular pathology. Constitutively active, Nox4 is also upregulated by AII, nonetheless it has recently received attention for its protective vascular properties [23]. Nox2 is associated with phagocytic respiratory burst activity, and expressed in endothelial cells. However studies looking at its role in atherosclerosis by specifically ablating it in ApoE-null mice failed to show any benefit [24]. Our finding that the NADPH oxidase activity brought about by L-NAME paralleled the induction of Nox1 suggests that this isoform is responsible for the activity we measured, and that it is dependent on the presence of PPAR*α*. Further, since NADPH oxidase is an established target for AII action, the concomitant alterations in several components of the aortic RAS observed in the Apoe-null mice are consistent with the notion that this system plays at least an ancillary role in the induction of NADPH oxidase in L-NAME treated ApoE-null mice, while this mechanism is not operative in the absence of PPAR*α*. Aortic ACE mRNA is much less expressed in DKO than in Apo-E mice, with or without L-NAME treatment. Furthermore, aortic renin and angiotensinogen mRNA expression are induced by L-NAME in the ApoE-null mice but not in the DKO mice, which parallels the absence of induction of aortic NADPH oxidase activity in this setting. In spite of the fact that aortic MCP1 mRNA expression significantly correlated with the degree of atherosclerosis, there was no further induction under L-NAME treatment in the ApoE-null mice. Such a result could have been expected given that it is also a target for AII. Although we cannot offer an explanation for this discrepancy, and perhaps different findings would have emerged had we measured the protein level, the fact that it was expressed at significantly lower levels in the DKO is reproducible [5] and needs to be emphasized.

In contrast to eNOS, which is widely expressed in the endothelium and is the main form of NOS in the normal vasculature, iNOS is barely detectable in normal vascular cells. Known to be induced by AII, iNOS produces large amounts of both NO and O_2_^−^, which by reacting together generate peroxynitrite. The latter further oxidizes LDL and uncouples eNOS. Thus iNOS is felt to exert a central role in the atherogenic process and is indeed abundant in atherosclerotic plaques [25, 26]. Moreover, genetic ablation of iNOS protected ApoE-null mice from atherosclerosis [27]. Consistent with the large difference in iNOS mRNA expression we observed between ApoE-null and DKO mice, amplification of mesangial iNOS expression by PPAR*α* agonists has been reported [28]. As L-NAME displays some specificity for eNOS [29], the low dose employed in the present study could have been particularly detrimental insofar as it inhibited endothelial NO production, while leaving iNOS activity unaffected.

Taken together, with the limitation that the expression data are based solely on mRNA levels, the data suggest that the presence of PPAR*α* is permissive for the expression of iNOS in the aorta of high fat-fed ApoE-null mice. This ensuing increase in oxidative burden could possibly underlie the difference in the extent of atherosclerosis we observed between the ApoE-null and DKO control animals.

**Figure 5:**
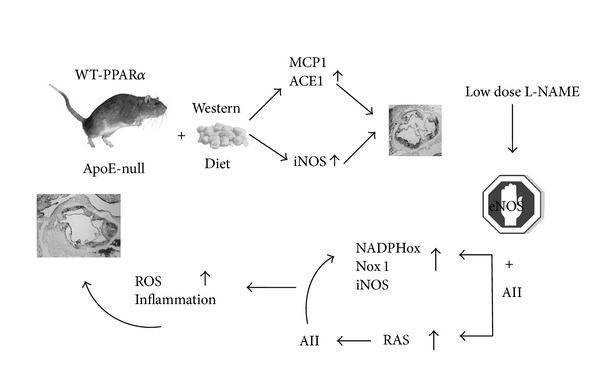
Proposed mechanism for the collusion of PPAR*α* and AII in the ApoE-null mouse with wild type (WT) PPAR*α* gene. The preferential eNOS activity inhibition by low dose L-NAME is suggested to alter the balance between AII and endothelium-derived NO, allowing amplification of the proatherogenic effect of unopposed AII action.

In summary, the findings suggest that, in the high fat-fed ApoE-null mouse, reduction of endothelial-derived NO unleashes PPAR*α*-dependent unopposed prooxidative and proatherogenic effects of AII, mediated both by NADPH oxidase through its Nox1 isoform, and by further induction of iNOS. We generated further evidence that not only is PPAR*α* central in the detrimental action of unopposed AII, but also that its presence may drive greater aortic RAS synthetic activity in response to decreased NO (a diagram summarizing the proposed mechanisms is given in Figure 5). We thus propose that, in the ApoE-null mice, absence of PPAR*α* mitigates the proatherogenic effect of reduced endothelium-derived NO supply.

## Figures and Tables

**Table 1:** Animals weights and systolic blood pressure at baseline and following treatment and biochemical measurements at the end of the study. The number of mice in each subgroup is shown in parentheses.

Parameter	ApoE-null males *n* = 26	ApoE-null females *n* = 23	DKO males *n* = 25	DKO females *n* = 19	*P *
Baseline weight (g)	23.6 ± 0.6	19.0 ± 0.6	26.3 ± 0.7	21.4 ± 0.7	<0.01 (males) 0.01 (females)
End weight control (g)	26.2 ± 0.8 (13)	21.6 ± 0.7 (9)	36.3 ± 1.6 (15)	29.0 ± 1.4 (10)	<0.0001*
End weight L-NAME (g)	27.7 ± 1.1 (13)	22.1 ± 0.5 (14)	32.8 ± 1.6 (10)	26.4 ± 0.6 (9)	<0.0001*
Baseline blood pressure^†^ (mm Hg)	106.6 ± 1.7		101.0 ± 2.1		NS^†^
End blood pressure control (mm Hg)	104.8 ± 2.9		104.1 ± 4.2		NS
End blood pressure L-NAME (mm Hg)	101.7 ± 1.7		102.9 ± 2.5		NS^†^
Cholesterol control (mg/dL)^‡^	737 ± 93^§^		1451 ± 147		0.001
Cholesterol L-NAME (mg/dL)	1021 ± 63		1026 ± 102		NS
Triglycerides control (mg/dL)	86.1 ± 6.4^§^		288.7 ± 47.9		<0.0001
Triglycerides L-NAME (mg/dL)	132.4 ± 14.5		260.5 ± 36.5		<0.0005

*For gender-specific comparisons.

^†^Blood pressure data are presented for males and females together as there were no differences between sexes. There were no differences between lines, treatment groups, or the time point at which blood pressure was measured.

^‡^Biochemical data are presented for males and females together as there were no differences between sexes in neither line.

^§^ *P* < 0.05 for comparison between ApoE-null control and ApoE-null with L-NAME.

**Table 2:** Aortic MCP1 and RAS components mRNA levels. Each group included 7–9 animals; while there were no differences between sexes, the breakdown by gender for each group is given in parentheses. Data are given as mean ± (SE). Data are expressed relative to the level in the ApoE-null control animals; thus, the Dunnett's posttest was chosen to follow the ANOVA.

Gene	ApoE-null control (4 M/4 F)	ApoE-null L-NAME (3 M/4 F)	DKO control (5 M/4 F)	DKO L-NAME (3 M/4 F)	*P* ANOVA
MCP1	1.0 (0.05)	1.02 (0.06)	0.6* (0.08)	0.5^†^ (0.13)	0.001
ACE1	1.0 (0.33)	0.55 (0.09)	0.27^†^ (0.09)	0.23^†^ (0.04)	0.005
Renin	1.0 (0.51)	2.57^‡^ (0.68)	2.0 (0.85)	1.68 (1.08)	NS
Angiotensinogen	1.0 (0.52)	2.25^‡^ (0.53)	1.26 (0.24)	1.0 (0.52)	NS
AT1-R	1.0 (0.24)	1.79 (0.78)	1.71 (0.42)	1.59 (0.34)	NS

*P < 0.05 versus control ApoE-null mice.

^†^ *P* < 0.01 versus control ApoE-null mice.

^‡^ *P* < 0.05 versus control ApoE-null mice by Student's *t*-test.
